# Toward the Value Sensitive Design of eHealth Technologies to Support Self-management of Cardiovascular Diseases: Content Analysis

**DOI:** 10.2196/31985

**Published:** 2021-12-01

**Authors:** Roberto Rafael Cruz-Martínez, Jobke Wentzel, Britt Elise Bente, Robbert Sanderman, Julia EWC van Gemert-Pijnen

**Affiliations:** 1 Department of Psychology, Health and Technology Faculty of Behavioural, Management and Social Sciences, Technical Medical Centre University of Twente Enschede Netherlands; 2 Department of Health and Social Studies Windesheim University of Applied Sciences Zwolle Netherlands; 3 General Health Psychology University Medical Center Groningen University of Groningen Groningen Netherlands

**Keywords:** eHealth, self-management, self-care, cardiovascular diseases, value sensitive design, values, content analysis

## Abstract

**Background:**

eHealth can revolutionize the way self-management support is offered to chronically ill individuals such as those with a cardiovascular disease (CVD). However, patients’ fluctuating motivation to actually perform self-management is an important factor for which to account. Tailoring and personalizing eHealth to fit with the values of individuals promises to be an effective motivational strategy. Nevertheless, how specific eHealth technologies and design features could potentially contribute to values of individuals with a CVD has not been explicitly studied before.

**Objective:**

This study sought to connect a set of empirically validated, health-related values of individuals with a CVD with existing eHealth technologies and their design features. The study searched for potential connections between design features and values with the goal to advance knowledge about how eHealth technologies can actually be more meaningful and motivating for end users.

**Methods:**

Undertaking a technical investigation that fits with the value sensitive design framework, a content analysis of existing eHealth technologies was conducted. We matched 11 empirically validated values of CVD patients with 70 design features from 10 eHealth technologies that were previously identified in a systematic review. The analysis consisted mainly of a deductive coding stage performed independently by 3 members of the study team. In addition, researchers and developers of 6 of the 10 reviewed technologies provided input about potential feature-value connections.

**Results:**

In total, 98 connections were made between eHealth design features and patient values. This meant that some design features could contribute to multiple values. Importantly, some values were more often addressed than others. CVD patients’ values most often addressed were related to (1) having or maintaining a healthy lifestyle, (2) having an overview of personal health data, (3) having reliable information and advice, (4) having extrinsic motivators to accomplish goals or health-related activities, and (5) receiving personalized care. In contrast, values less often addressed concerned (6) perceiving low thresholds to access health care, (7) receiving social support, (8) preserving a sense of autonomy over life, and (9) not feeling fear, anxiety, or insecurity about health. Last, 2 largely unaddressed values were related to (10) having confidence and self-efficacy in the treatment or ability to achieve goals and (11) desiring to be seen as a person rather than a patient.

**Conclusions:**

Positively, existing eHealth technologies could be connected with CVD patients’ values, largely through design features that relate to educational support, self-monitoring support, behavior change support, feedback, and motivational incentives. Other design features such as reminders, prompts or cues, peer-based or expert-based human support, and general system personalization were also connected with values but in narrower ways. In future studies, the inferred feature-value connections must be validated with empirical data from individuals with a CVD or similar chronic conditions.

## Introduction

### The Promise of eHealth for Self-management Support

Self-management can be broadly defined as an individual’s ability to manage the symptoms, treatment, physical and psychosocial consequences, and lifestyle changes inherent in living with a chronic illness [1]. In 2005, the influential psychologist Albert Bandura [2] characterized self-management as “good medicine” and went even further, stating that “if the huge benefits of these few habits were put into a pill, it would be declared a scientific milestone in the field of medicine.” Such a milestone would certainly lead to a much-needed reduction of the alarming burden on health care systems worldwide caused by the increasing amount of chronically ill individuals, many of them with a cardiovascular disease (CVD) [3].

Obviously, there is not yet—and perhaps there will never be—a “pill” that prompts individuals to actively engage in the maintenance, monitoring, and management of their own health. The reality is much more challenging, as performing self-management entails the enactment of multiple behaviors and a continuous confrontation with barriers and competing interests [4]. For example, stroke survivors can be overwhelmed by the physical and cognitive efforts required by rehabilitation programs and by other sudden changes to their lifestyles, leading them to feel as if they have “lost control” over their life.

Although not a “pill,” the use of digital technologies to support health, well-being, and health care holds high promise. Such an approach is better known by the term of electronic health or eHealth [5]. Specifically, technologies such as smartphone applications and internet-enabled monitoring devices have been proposed as tools that can support self-management [6,7]. Among other things, eHealth promises to facilitate tasks and provide personalized information, feedback, or cues to action. eHealth technologies have, in fact, already shown positive results in terms of supporting patients in the management of chronic conditions, including CVD [6-13].

### Realizing the Promise of eHealth Through Value Sensitive Design

Despite their promising results and recognized potential, eHealth technologies that aim to support self-management have come across multiple challenges. One of the most important obstacles is the fluctuating motivation of individuals to actually perform self-management [9,10]. As a result, when motivation is low, eHealth technologies can become an added burden [14]. To overcome that barrier, multiple calls have been made to design eHealth in a way that better aligns with the underlying needs of individuals [6,7,10,15]. One key proposal is that eHealth technologies should be personalized in a way that taps into a more powerful source of motivation: values. To realize this, eHealth technologies should be designed in a way that strengthens patients’ values and fulfills their needs. For instance, patients who highly value social interactions could be motivated through eHealth features that facilitate communication with peers, friends, or the health care team.

In fact, the need to meet patient values through the design of technologies has led to the development of novel methodologies and theoretical approaches. One of these approaches is value sensitive design, which serves as both a theoretical and methodological framework that seeks to integrate values into design work [16]. Value sensitive design ensures that the design of technologies accounts for values in a principled and comprehensive manner, through integrative and iterative methodologies that include conceptual, empirical, and technical investigations [16]. Conceptual investigations can focus on the philosophical analysis and specification of value constructs (eg, the value of “feeling in control” or the value of “feeling supported by others”). Meanwhile, technical investigations can take the analysis further and design technologies using the identified values as assessment criteria (eg, how do wearable technologies meet the value of “feeling in control over life?”). Finally, empirical investigations can evaluate the process of a particular design or context use (eg, a formative evaluation of technologies to assess if and how they contribute to patient values).

### Conceptualizing Values for eHealth Design

In the value sensitive design framework, a value refers to “what a person or group of people considers important in life” [16]. In eHealth, this could translate to a life ideal or important interest, related to health or well-being, that individuals could pursue or meet with the help of technologies [15]. This paper uses the terms “values” and “patient values” interchangeably. Moreover, this paper uses the term “connection” to refer to a potentially positive relationship between a specific technology—or one of its design features—and a patient’s value that leads to an increase or maintenance of motivation (eg, a self-monitoring feature might be “connected” to the value of “feeling safe and stable”). Other terms used in scientific works talk about how technologies or design can “contribute,” “meet,” “support,” or “honor” values. These verbs are all understood to refer to the same relationship.

As mentioned before, incorporating values into technologies can entail multiple integrative and iterative steps. For instance, value specification precedes value sensitive design. Value specification is the identification of the most important values for stakeholders of eHealth (eg, end users such as individuals with a CVD) [17]. Holistic approaches to eHealth development and design, such as the one promoted by the Center for eHealth Research (CeHRes) Roadmap [18], stress the importance of identifying the diverse and often conflicting values and concerns that different stakeholders have (eg, what does a patient value in health and life and thus expect to be helped with through eHealth?). This raises a fundamental question: What values must be considered to design effective support for the values of individuals with a CVD? A previous investigation by authors of this study directly addressed this question [19]. Concretely, an interview study integrated a list of 11 values of patients with a CVD [19]. Then, as a follow-up study, the list of values was revised and empirically validated through a survey with members of a patient association in the Netherlands, constituted by individuals who have attended or are still attending a cardiac rehabilitation program [19]. Therefore, there are already available data establishing a set of potential values of importance for individuals diagnosed with a CVD.

### Connecting Values With eHealth Technologies and Design Features

Importantly, the value sensitive design framework also presupposes that a given technology is more suitable for certain activities and more readily supports certain values, while rendering others more difficult to realize [16]. Therefore, it suggests that it all depends on the “features” or “properties” that people design into technologies. In this study, the term “design feature” is used to define any clearly identifiable property of a technology that serves a specific function and is proposed to help achieve an overarching aim. Given such a definition, design features could be functional or visual properties, underlying technical mechanisms, as well as recognizable “building blocks” such as behavior change techniques [20] and persuasive design strategies [21]. Furthermore, this study defines an eHealth technology as a (set of) technological instrument(s), such as a mobile app, that is specifically developed to support well-being, health, or health care [5]. In contrast, an eHealth intervention is defined as the full package and procedures that describe how a specific eHealth technology intervenes to support well-being, health, or health care [5]. The former concept is favored because the focus of this study is design features of technologies that are at different stages of development (eg, from high-fidelity prototypes to systems that have already been implemented and evaluated).

In light of the aforementioned information and given the numerous examples of eHealth technologies that exist, it is plausible that several values have already been met by their design features. However, to the best of our knowledge, the connection between specific design features and patient values has not been directly investigated in previous studies. Therefore, it is necessary to advance the understanding about how technologies can best support the values of individuals. This knowledge can be uncovered through what the value sensitive design framework calls “technical investigations,” which are studies that focus on how existing technological properties and underlying mechanisms support or hinder values [16]. In this way, technical investigations could help advance knowledge about what works, for whom, and why in terms of CVD self-management [22]. Consequently, evidence on the most effective technological properties and mechanisms could be translated into practical guidelines for the development and design of future eHealth technologies.

As empirical knowledge about the values of individuals with a CVD already exists, what is needed is a set of technologies that can be investigated with the aforementioned aim in mind. To that end, the outcomes of a recent systematic review that identified and analyzed multiple eHealth technologies for CVD self-management could be used [23,24]. The review analyzed technologies with sufficient and substantial information about their objectives and design (ie, their design features). Thus, information about the design features of existing eHealth technologies is also readily available for the purposes of this investigation.

### Aim

This study sought to connect a set of empirically validated values of patients diagnosed with a CVD with existing eHealth technologies and their design features. By doing so, the findings of the study aimed to be a foundation for new hypothetical assumptions that contribute to value sensitive eHealth design and that could be validated in future empirical studies.

Content analysis is proposed as a suitable method to meet this aim because it allows making replicable and valid inferences from texts or other meaningful matter to the contexts of their use [25]. As a scientific tool, content analysis can provide new insights, increase the understanding of particular phenomena, or inform practical actions [25]. In short, content analysis offers a sound and verifiable method that can connect patient values with multiple and distinguishable eHealth design features. Following what has been issued in the previous sections, this research follows a patient-centered design approach to focus on the main drivers of patients’ needs and concerns: their values. The research question is: *What eHealth design features can be connected with the values of patients with a CVD?*

## Methods

### Overview

To meet the study aims, the research team conducted a content analysis [25]. The content analysis consisted of 3 stages: preparation, organization, and analysis and reporting [26]. The main researcher (RRCM) conducted the preparation stage by collecting and setting up the data to analyze the eHealth design features [26]. Next, 3 researchers (RRCM, JW, and BEB) performed the organization stage independently by deductively coding the data [26]. Finally, all researchers contributed to the reporting stage, consisting of displaying the results according to the selected approach and categorization scheme [26].

### Preparation

The preparation stage aimed to identify design features of existing eHealth technologies and to describe them in a format that facilitated their analysis. To identify eHealth design features for the study, RRCM revised and expanded the data extracted about 10 eHealth technologies during a previous literature systematic review [23,24]. Additionally, RRCM searched for newer publications of all technologies through reference tracking of the included papers. Importantly, RRCM extracted both descriptive and contextual information about each eHealth design feature. Descriptive information could be a clear textual description of the design feature (eg, what it does or intends to do according to the publication) and a figure or picture of it (when available). In contrast, contextual information could be the name of technologies, their main characteristics, their target group, and any specific objectives. RRCM integrated all descriptive and contextual information about each eHealth design feature in separate Microsoft PowerPoint slides. For example, the Engage mobile application included 5 design features [27]: log, hint/facts, goal, progress report, and deck of cards.

At this stage, RRCM noticed and began to group the design features of different technologies according to their similar characteristics or functions. For example, the “log” feature of the Engage technology [27] is similar to the “assessment” feature of the HeartMapp [28] technology, in the sense that they both facilitate self-reporting of symptoms and other self-management behaviors. The researchers finally agreed on the final grouping of design features at the analysis and reporting stages (as described in the following sections). In this way, both descriptive and contextual information facilitated a better comprehension of eHealth design and its features. In total, the study analyzed 70 design features from 10 different CVD eHealth technologies. Multimedia Appendix 1 presents a detailed overview of the included technologies and their design features.

### Organization

The organization stage aimed to connect a list of 11 empirically validated patient values to the eHealth design features by means of deductive coding. A usability study and a follow-up survey study generated and validated the list of values [19]. The first study consisted of 10 interviews within the context of patients’ usability tests with the online BENEFIT Personal Health Platform, which aims to support the adoption and maintenance of healthy lifestyles [19]. The second study distributed an online survey to panel members of Harteraad, a Dutch patient association for cardiac diseases (in total, the survey had 710 respondents) [19]. In this survey, the respondents rated the values identified in the first study according to their importance for themselves, which aimed to estimate relevance and generalizability of the values in a larger population. To prepare the codebook for this study, BEB and JW translated the list of values from the Dutch language into English. Table 1 presents the list of values in their final form as the codebook for this study.

**Table 1:** Codebook with list of patient values and their definitions.

Number	Value label	Value definition
1	To have confidence and self-efficacy in treatment and ability to achieve goals	Having confidence in the doctors and the treatment they prescribe or having the feeling that patients are capable of following the treatment plan or have the ability to achieve their goals
2	To be seen as a person rather than a patient	Not constantly feeling that they are a patient with a disease but also still being able to be a human without their illness
3	To not feel fear, anxiousness, or insecurity about their health	Not having to worry about their physical condition, being provided coping strategies or information that helps them feel safe or less anxious
4	To preserve a sense of autonomy over their life	Having a feeling of being in control of their life (eg, being able to make their own decisions)
5	To receive social support	Feeling heard, supported, and understood by the people that surround them (eg, family and friends) and having the feeling that they have somewhere or someone to go to when they need a sympathetic ear (eg, via a virtual coach or a chat)
6	To have or maintain a healthy lifestyle	Maintaining or changing their lifestyle in such a way that new incidents are prevented and they (re)gain health
7	To have an overview of personal health data	Having a central source where they have insight into their personal health data or condition (eg, measured values or any insights into physical and mental well-being and health)
8	To perceive low thresholds to access health care	Being helped or treated quickly and easily, at a health care organization or at home; being facilitated to manage their own disease and take action
9	To be extrinsically motivated to accomplish goals or activities (related to health/lifestyle)	Being extrinsically motivated to do or accomplish things, such as their treatment or activities for a healthy lifestyle (eg, via social pressure)
10	To have reliable information and advice	Having understandable, relevant information and advice that is scientifically proven and recommended by the clinical team (ie, evidence-based information)
11	To receive personalized care	Receiving a personal approach in which their opinion and preferences are taken into account (eg, personalization or tailoring of treatment choices or features)

RRCM, BEB, and JW independently performed the coding of the eHealth design features. All coders are experts in eHealth research and development, having overall conducted various studies focused on eHealth design and evaluation involving multiple stakeholders’ perspectives (eg, end users such as patients or expert stakeholders such as health care providers). The researchers first conducted a pilot of the coding using design features of a technology that was not included in the systematic review (the Care4myHeart app [29,30]). Minor adjustments were made to the codebook based on the resulting discrepancies. During coding, each researcher could characterize the connection between a specific design feature and a patient value as follows: (1) “Yes,” if the design feature directly and clearly accomplishes or contributes to a value; (2) “Maybe,” if the design feature accomplishes or contributes to a value only indirectly or if the information is unclear; and (3) “No,” if the design feature clearly does not accomplish or contribute to a value.

In addition to the deductive coding stage, RRCM invited authors of publications related to the included technologies via email to fill in a self-assessment form that asked about the relationship between their technology and the list of patient values. The self-assessment form posed 2 questions: (1) “Do you consider that your intervention accomplishes or contributes to any of the patient values listed below?” and (2) “When applicable, can you specify which feature or part of the intervention you consider seeks to accomplish or contribute to the corresponding patient value?” Finally, respondents could also freely state if other patient values outside the list provided were considered targets of the technology. In this way, it was expected that authors could link their technology and one or multiple design features to one of the values in the codebook. Multimedia Appendix 2 presents the self-assessment form that authors were invited to fill in. During the coding stage, the research team was blinded to any self-assessment sent by the researchers or developers of technologies.

### Analysis and Reporting

To analyze the results, simple agreements (percent agreements) and the interrater reliability resulting from the deductive coding were calculated. Krippendorff alpha (KALPHA) was used as the measure of interrater reliability because, among other things, it takes into account the expected disagreement and not only the observed disagreement [25,31]. Values of KALPHA range from 0 to 1, where 0 is perfect disagreement and 1 is perfect agreement. Although it depends on the context, an alpha >0.80 is usually ideal, and a minimum level of acceptance is typically 0.667 [25].

Although independent coding performed by the research team led the search for potential connections, the input received from researchers and developers of technologies could support the identification when full agreement was not achieved. Therefore, the positive identification of a potential connection had to meet 1 of 2 criteria. The first and main criterion was to have full agreement on a connection among the 3 coders (ie, 3 out of 3 agreed on a feature-value connection). However, a potential connection was also recorded when the input by researchers and developers of technologies suggested it, as long as there was also partial agreement between coders (ie, 2 out of 3 agreed independently on a feature-value connection).

To report the results, the connections were first summarized at the level of the technologies. This first summary is reported because it is important to understand—and later to discuss—the surrounding context of the design features, which could have a relationship with their potential connections with patient values (eg, the intended goals of technologies that led design choices). Next, the design features that were connected with values were grouped according to their objectives and functionalities (eg, grouping different design features that relate to “self-monitoring” support, as with the previously mentioned “log” and “assessment” design features). By grouping specific design features according to their common characteristics, it was easier to identify potential differences in their design and their potential connections to values. For example, 2 different self-monitoring support design features could still be distinct enough that one could potentially contribute directly and clearly to a value while another one does so indirectly. This meant that some types of design features could entail both direct and indirect pathways toward a value. When relevant, some outstanding design features were textually described (eg, features that contributed to largely unaddressed values).

## Results

### Deductive Coding

In total, 70 design features from 10 different eHealth technologies were used for the content analysis (see Multimedia Appendix 1 for the full overview). To recall, each design feature was coded according to its potential connection with 11 different values (as “Yes,” “Maybe,” or “No”). Table 2 presents a summary of the percent agreements that resulted from the independent deductive coding. As can be observed in Table 2, 41 direct and clear connections between design features and patient values were identified in this way (ie, the ones with full agreement on “Yes”). In addition, 4 pairings were characterized as indirect or unclear (ie, the ones with full agreement on “Maybe”).

The KALPHA coefficient for all data was 0.4536 (95% CI 0.4087-0.4978), which is low (0.667 is typically the minimum acceptable level [25]). KALPHA was computed using an ordinal measurement level that treated the potential connection between a design feature and a patient value as increasing from “No” (0) to “Maybe” (1) and “Yes” (2).

At the start, as can be seen in Table 2, 44 connections (41 “Yes” and 4 “Maybe”) were identified through deductive coding. However, after integrating the input of researchers and designers of the reviewed technologies, the inferred connections between eHealth design features and patient values increased up to a total of 98 connections. Of the 45 researchers invited to complete the form, 6 individuals returned it (6 more also responded but redirected the request to a co-author who ultimately responded). Each form received related to a different technology; therefore, input was received for 6 of the 10 reviewed technologies: Engage [27], HeartMapp [28,32,33], HOME BP [34-38], PATHway [39,40], SMART-PSMS [41-46], and SUPPORT-HF [47-50]. For the remaining technologies, the authors either declined the invitation or did not respond after several reminders: MedFit [51-53], MyHeart [54-56], SMASH [57-62], and Mock-Up by Baek et al [63].

**Table 2:** Summary of percent agreements from deductive coding of 70 eHealth design features according to the potential connection with 11 different patient values, resulting in 770 possible connections between a design feature and a patient value.

Level of agreement			Results, n (%)
Connections with *full* agreement (ie, 3 out of 3)			502 (65.2)
**Responses for connections with *full* agreement (ie, 3 out of 3)**			
	Yes	41 (8.2)	
	Maybe	4 (0.8)	
	No	457 (91.0)	
Connections with *partial* agreements (ie, 2 out of 3)			209 (27.1)
**Responses for connections with *partial* agreements (ie, 2 out of 3)**			
	Yes	48 (23.0)	
	Maybe	10 (4.8)	
	No	151 (72.2)	
*Null* agreement (ie, 0 out of 3)			59 (7.7)

### Contributions of Existing eHealth Technologies to Patient Values

The design features reviewed in this study were not created in isolation. Their surrounding context was an overarching eHealth technology with specific goals that led design choices. Because such context is important, it is also relevant—although not the focus of the study—to report the identified connections between eHealth technologies and patient values. The 98 connections suggest that some of the values are addressed by a majority of the 10 eHealth technologies. For instance, all of the technologies were connected with the patient value of “having or maintaining a healthy lifestyle.” Similarly, the following values were connected with 8 different technologies: “having an overview of personal health data,” “having reliable information and advice,” “being extrinsically motivated,” and “receiving personalized care.” Less frequently, the “perceiving low thresholds to access health care” value was connected with 6 different technologies.

In contrast, other values connected with only a minority of the reviewed eHealth technologies. For instance, only 3 of 10 technologies were connected with the patient value of “receiving social support”: PATHway [40], MedFit [51-53], and HOME BP [34-38]. Likewise, only 3 different technologies were connected with the patient value of “not feeling fear, anxiousness, or insecurity about health”: SMASH [57,61], HOME BP [34-38], and SUPPORT-HF [47,49]. Only 2 technologies were connected with the patient value of “preserving a sense of autonomy”: Engage [27] and the SMART PSMS [43,44]. Only the “On-screen positive reinforcement” design feature of the PATHway technology was connected with the patient value of “having confidence and self-efficacy in the treatment and the ability to achieve goals” [39,40]. Similarly, only the “culturally-attuned motivational and reinforcement SMS messages” design feature of the SMASH technology was connected with the patient value of “being seen as a person rather than a patient” [58,61,62].

### Contributions of eHealth Design Features to Patient Values

The eHealth design features could be grouped according to their similar objectives and functionalities (ie, what they aim to do and how they try to do it). In total, the analysis identified 13 distinguishable “types” of design features: educational support, self-monitoring support, behavioral assessment support, behavioral planning support, behavioral performance support, feedback on monitored data, feedback during behavior performance, motivational incentives, prompts or cues, reminders, peer-based support, expert-based support, and the personalization of the system’s design features. Textbox 1 presents descriptions and examples of the types of eHealth design features.

**Types of design features of eHealth technologies that support self-management of cardiovascular disease (CVD).**

- Educational support: Features that enable the patients to access educational materials on various topics (eg, the “Heart Failure (HF) Info” feature of HeartMapp [28,32,33]); educational information could be presented with text, audio, or videos.
- Self-monitoring support: Features that facilitate the patient’s monitoring of various types of data (eg, the “log” feature of Engage [27]), for instance, monitoring symptoms, weight, or self-management behaviors.
- Behavioral planning support: Features that facilitate selection and action-planning of health maintenance behaviors (eg, the “goal” feature of Engage [27]), for instance, to decide when and how to exercise based on long-term goals that were either self-set or agreed upon with health care providers.
- Behavioral performance support: Features that provide information, guidance, or support for the actual performance of health maintenance behaviors (eg, the “exercise” feature of MedFit [51-53]), for instance, an animated deep breathing practice or a list of guided exercise classes; the features can include real-time feedback or self-evaluation options (eg, rating performance or intensity).
- Behavioral assessment support: Features that assess a patient’s readiness to change a selected behavior (eg, PATHway’s “behavioral change assessment” and “good habits visualization” [40]); they can lead to a visual display of risk factors or recommended priorities for behavior change.
- Feedback on monitored data: Features that present graphs, charts, or written reports of a patient’s data over time (eg, “statistics/stats” feature of HeartMapp [28,32,33]); the data can be about symptoms, behaviors, or the progress toward a desired performance.
- Feedback during behavior performance: Features that provide real-time feedback during the performance of health maintenance behaviors (eg, the “on-screen positive reinforcement” feature of PATHway [39,40]), for instance, to incentivize the correct execution of physical rehabilitation exercises.
- Motivational incentives: Features that incentivize engagement with the technology by using metaphors such as “missions,” “medals,” or “cards” (eg, the “deck of cards” feature of Engage [27]); they can be personalized according to a prescribed treatment, self-set goals, or automatic analyses of data collected.
- Cues: Features that provide prompts or cue to actions (eg, the “behavior change notifications” feature of PATHway [40]); they are directed to specific behaviors and can be personalized to a patient’s preferences.
- Reminders: Features that provide reminders to facilitate adherence to medication (eg, the “medication tray reminder signals” of SMASH [59-61]); they can include the demand of an action or a request for additional input such as a reason for not conducting the behavior (eg, report the intake of medication as prescribed or a reason for skipping it).
- Peer-based human support: Features that facilitate interaction with peers (eg, the “multiplayer class” feature of PATHway [40]), for instance, through online platforms that allow data comparison between individuals or make it possible to plan activities with others.
- Expert-based human support: Features that focus on the interaction or involvement of health care providers (eg, the “contact” feature of SUPPORT-HF [47-49]); they can include a communication channel with an expert or support team and be linked to a clinical team module or a back-end alarm system that prompts interaction.
- System personalization features: Features that aim to (de-)activate the system’s modules based on individual needs (eg, the “remote system refinements and features activation” feature of SUPPORT-HF [48,49]); personalization can occur at the initial introduction of the technology or as a response to the evolving situation of the individual.

**Figure 1:**
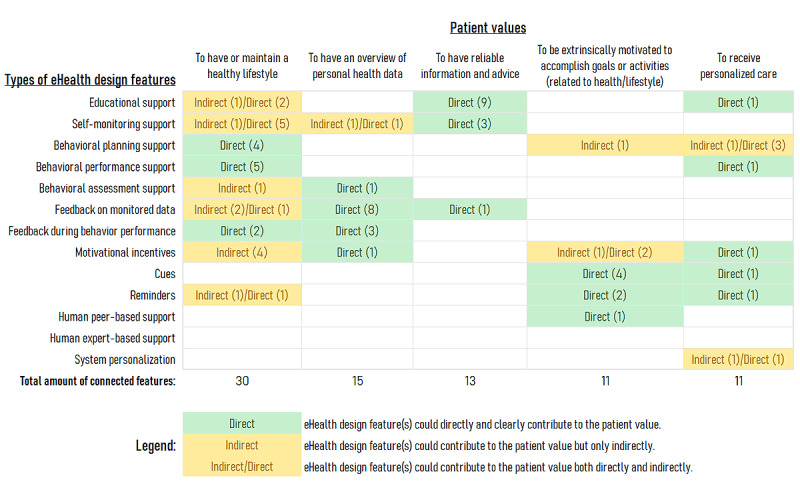
Overview of the types of eHealth design features that were most frequently connected with values of patients with a cardiovascular disease.

**Figure 2:**
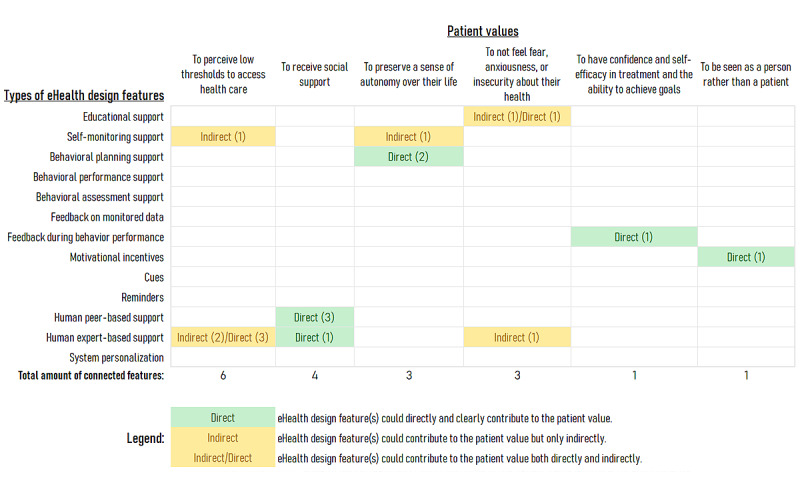
Overview of the types of eHealth design features that were least frequently connected with values of patients with a cardiovascular disease.

The results of the content analysis revealed that different (types of) design features from existing eHealth technologies could be connected with values of patients with a CVD. Figures 1 and 2 present overviews of how the different types of eHealth design features connected with one or more patient values. Both figures summarize the cases where at least one specific design feature connected with a value and mark whether that connection was inferred to be direct or indirect. To recall, a direct connection referred to a clear and potentially positive relationship between a design feature and a patient value, leading to an increase or maintenance of motivation for self-management. In contrast, an indirect connection referred to an instance where the positive relationship required some assumptions to be made on behalf of the research team (eg, because information about a design feature’s functionality was unclear or unavailable). Moreover, both figures also show that, in some cases, design features within the same category could have different connections (ie, one direct and another indirect).

Figure 1 summarizes the patient values most frequently connected with the eHealth design features analyzed in this study. As can be seen in Figure 1, 5 of the 11 patient values were extensively connected with multiple design features with distinct characteristics and objectives. An apparent exception is the “to have reliable information and advice” value, which was connected with 3 types of design features (educational support, self-monitoring support, and feedback on monitored data). However, even in that case, the total amount of specific design features was relatively high (13 in total). Beyond frequencies, Figure 1 also visualizes potential clusters of design feature types in relation to patient values. For instance, several features providing feedback on monitored data connected with the value of “having an overview of personal health data.” Likewise, motivational incentives, cues, and reminders most frequently connected with the value of “being extrinsically motivated.”

In contrast to the aforementioned results, Figure 2 summarizes the patient values least frequently connected with the eHealth design features analyzed in this study. Figure 2 shows that, for the remaining 6 patient values, the amount of connected design features is fewer, also varying less in their functionalities or objectives. In comparison with Figure 1, the values presented in Figure 2 connected only, at most, with 2 different types of eHealth design features. Beyond mere frequencies, Figure 2 shows that both human peer–based and expert-based support clustered toward a couple of the values in Figure 2. Namely, the values of “perceiving low thresholds to access health care” (5 specific features) and “receiving social support” (4 specific features). The rest of the values in Figure 2, however, connected only to a maximum of 2 specific features. Finally, the values of “having confidence and self-efficacy” and “being seen as a person rather than a patient” connected only with a single feature each.

## Discussion

### Principal Findings

This study sought an answer to the research question “what eHealth design features can be connected with the values of patients with a CVD?” To approach an answer, the study explored potential connections between 11 empirically validated values of patients diagnosed with a CVD and 70 design features of 10 existing eHealth technologies that aim to support this population. In total, 98 connections—both direct and indirect—were inferred between the design features and the values included in the analysis. On the one hand, some design features connected with multiple values. On the other hand, some values were less frequently connected, with a couple remaining largely unaddressed.

Principally, the results of the study show that design features of existing eHealth technologies could already be connected with values of individuals with a CVD (see Figures 1 and 2). The findings add up to the general literature about value sensitive studies of chronically ill populations and the design of self-management eHealth solutions. The connections between design features and values inferred by this study are still hypothetical, but the knowledge generated can be used to suggest new approaches for the development of personalized and tailored eHealth. The following discussion centers on the arguments that underlie outstanding cases among the 98 inferred connections, as well as some of their potential applications to the design of eHealth for self-management support.

### Inferred Connections Between eHealth Design Features and Patient Values

#### Supporting Patients Who Value “a Healthy Lifestyle”

It comes arguably without surprise that the most frequently connected patient value was “to have or maintain a healthy lifestyle” (see Figure 1). Design features such as goal setting, suggestions, or reminders have been identified as key components of eHealth technologies that aim to promote healthy lifestyles [64]. Figure 1 reflects a similar variety in the types of eHealth design features connected with this value (eg, all forms of behavioral support). Outstandingly, design features related to behavioral planning support, behavioral performance support, and the provision of feedback during behavior performance directly connected with this value. However, the analysis identified only 2 examples of real-time feedback features during performance. Specifically, the “on-screen positive reinforcement” feature of PATHway [38,39] and the “upper-limb rehabilitation” feature of the SMART PSMS stroke module [46]. Similarly, the PATHway “behavioral change assessment” feature stood out as a way to potentially and indirectly honor this value [40]. The aforementioned features could represent untapped design opportunities to support individuals who highly value the maintenance of a healthy lifestyle (full details and references to specific features can be found in Multimedia Appendix 1).

#### Supporting Patients Who Value “an Overview of Personal Health Data”

The study also connected several eHealth design features with the value of “having an overview of personal health data” (Figure 1). These included all types of feedback provision but also self-monitoring support, behavioral assessment support, and even motivational incentives. That the agreed connections went beyond the “typical” feedback features (eg, statistics charts) could arguably hint toward ways to resolve the challenges reported by patients for the *sensemaking* of their health data [65,66]. Sensemaking is considered the explicit and effortful approach of individuals to analytically engage with a situation, in order to construct explanations that allow them to select appropriate actions [65]. For example, the “good habits visualization” feature of PATHway [40] is a behavioral assessment feature that not only delivers an overview of data but also suggests areas that need to be improved. Similarly, the self-monitoring features connected with this value included a follow-up overview of monitored data. Specifically, the “self-management” feature of mock-up by Baek et al [63] directly provides an overview of data, while the “log” feature of Engage [27] indirectly does so by requiring a few actions to access one. The “walking re-education and foot placement” feature of the SMART PSMS stroke module is the single motivational incentive feature connected with this value [44,45]. The overview provided by this feature emphasizes a feeling of progress and reward [45]. Studies from the sensemaking perspective support the notion that data-driven features can engage patients in different ways, by providing external motivational incentives, facilitating goal setting, or, in a lesser degree, allowing open exploration of their health data (ideally triggering sensemaking) [67,68].

#### Supporting Patients Who Value “Reliable Information and Advice”

Unsurprisingly, multiple educational support features connected with the value of “having reliable information and advice” (Figure 1). Additionally, self-monitoring and monitored data feedback features connected with this value by guiding correct monitoring procedures and providing quick practical advice. For example, the “assessment” feature of HeartMapp goes beyond just self-monitoring support by classifying patients according to safety levels and delivering behavioral actions [28]. Importantly, some features connected also with other less frequently addressed values, such as “not feeling fear, anxiety, or insecurity” or “having confidence and self-efficacy.” The struggles of patients in their transition from hospital-based care to self-managing at home are widely acknowledged [69]. The ability to access reliable information and advice during and after this transition could underlie the aforementioned feature-value connections but also a relation between patient values.

#### Supporting Patients Who Value “Extrinsic Motivation”

The study also connected multiple eHealth design features with the value of “being extrinsically motivated to accomplish goals or activities related to healthy lifestyles” (Figure 1). Cues, reminders, peer-based support, and motivational incentives directly connected with this value. These connections could be supported by the available evidence on the positive effects of social support [70] and of features that prompt immediate behavioral action [71], remind patients about key activities [29], or aim to motivate self-management in general [57,72]. In this regard, the “culturally-attuned motivational and reinforcement SMS messages” of the SMASH technology stood out because it also directly connected with other values, including the least frequently addressed value of “being perceived as a person rather than a patient” [57,58,61]. Finally, the “goal” feature of Engage was the only behavioral planning feature indirectly connected with the “extrinsic motivation” value [27]. The argument for the indirect connection is its integration with the “deck of cards” motivational feature [27].

#### Supporting Patients Who Value “Personalized Care”

As with the previous cases, the study connected several eHealth features with the value of “receiving personalized care” (Figure 1). These included educational support features; behavioral planning and performance support; and motivational incentives, cues, and reminders. As an example, the “optional lifestyle changes” educational feature of HOME BP allows patients to personally request additional content [34-38]. Alternatively, the “exercise” feature of MedFit automatically updates the list of guided exercise classes based on the evaluation of classes performed earlier [51-53]. Outstandingly, 2 overarching system personalization features connected with this value. On the one hand, the “my stroke” feature of the SMART PSMS permitted the customization of the system during its deployment, with the involvement of both the patient and health care provider [43,44]. On the other hand, the “remote system refinements and features activation” of SUPPORT-HF connected indirectly because the personalization seemed to be exclusively controlled by clinicians [48,49]. Both features exemplify what appear to be still untapped opportunities in terms of modular customization of eHealth technologies for individual cases.

#### Supporting Patients Who Value “Low Thresholds to Health Care”

In contrast to the previous values, only 5 human expert–based support features and a single self-monitoring support feature connected with the value of “perceiving low thresholds to access health care” (Figure 2). The connections with expert-based support features align with literature highlighting the irreplaceable role of health care providers, especially when it comes to remote support [66,73]. In this regard, front-end support features permitting the patients to trigger, request, or receive advice from professionals connected directly with this value. For example, the “contact” feature of SUPPORT-HF allows patients to contact the support team [47,48,50]. In comparison, back-end features exclusively available to health care providers connected only indirectly, for example, the “clinical team module” of the HeartMapp application [33]. Standing on its own, the “today’s exercise” self-monitoring feature of the SMART PSMS stroke module also connected indirectly with this value [43,45]. This specific connection was argued on the integration of a preliminary check of symptoms and mood, which, if necessary, prompts patients to call the hospital for assistance before initiating exercises [43,45].

#### Supporting Patients Who Value “Social Support”

Expectedly, 3 peer-based support features connected with the value of “receiving social support” (Figure 2). PATHway’s “multiplayer class” and “calendar for events/exercise” features [40] as well as MedFit’s “social interaction” feature connected directly with this value [51-53]. Perhaps more surprising in this case is that the expert-based “behavioral support (via health care provider)” feature of HOME BP connected with this value [34-38]. This feature gives patients the option to request face-to-face or telephone-based behavioral support for self-monitoring and lifestyle modifications [34-38]. The underlying argument for this connection was the implementation of a training protocol for caregivers called “congratulate, ask, reassure, encourage” or CARE [34-38]. Although patients’ families and peers are typically the expected sources of social support, a recent study acknowledged that health care providers can also play significant roles in this regard [74].

#### Supporting Patients Who Value “a Sense of Autonomy”

This study only connected 3 eHealth design features with the value of “preserving a sense of autonomy” (Figure 2). The “goal” feature of Engage [27] and the “my exercises” feature of the SMART PSMS stroke module [43,44] connected directly by allowing patients to create their own self-management action plans. Indirectly connected, Engage’s “log” self-monitoring feature allows patients to select and record the performance of activities based on a predetermined set of recommended actions [27]. Supporting this connection, recent works ascertained how the support for autonomy can also promote the patients’ individual responsibility for their own care [71,73]. The aforementioned features exemplify how eHealth might be able to promote autonomy, that is, by providing options and thus avoiding fixed or generic recommendations for self-management.

#### Supporting Patients Who Value “Not Feeling Fear, Anxiety, or Insecurity”

The study directly connected only 1 eHealth design feature with the value of “not feeling fear, anxiousness, or insecurity about health” and 2 more indirectly (Figure 2). The “education about medication titration” feature of HOME BP connected directly because it addressed potential concerns about the side effects of medication [34-38]. The “how to keep healthy” educational feature of SUPPORT-HF connected indirectly by its presentation of videos depicting other patients’ stories [47,49]. The “clinical inertia alarms (to health care providers)” feature of SMASH [57,61] also connected indirectly. In this regard, a study has reported how awareness of such links with health professionals can generate feelings of safety in patients [75]. The small amount of features connected with this value is worrying in consideration of the feelings of fear, anxiety, and hopelessness that are commonly reported by patients with a CVD [69,76]. Therefore, it seems important that future eHealth technologies aim to assist the patient’s control over these emotions. Although not reviewed by this study, there are some design examples that go beyond those already mentioned, such as feedback during behavior performance based on optimal training zones identified through heart rate monitoring (eg, during cycling [77]).

#### Supporting Patients Who Value “Confidence in Treatment and for Goal Achievement”

The “on-screen positive reinforcement” of PATHway is the only feature connected with the value of “having confidence and self-efficacy in the treatment and the ability to achieve goals” [39,40] (Figure 2). This specific finding could represent an important gap in eHealth design, as self-efficacy is known to be a key influencing factor for self-management behaviors [78,79]. Future eHealth technologies could attempt to integrate principles of evidence-based approaches such as motivational interviewing [80]. Alternatively, it could be explored why previous design approaches seem to fall short in boosting self-efficacy, that is, because a recent scoping review of digital games aiming to support CVD self-management concluded that they failed to improve the self-efficacy of patients [81].

#### Supporting Patients Who Value “Being Seen as a Person Rather Than a Patient”

Finally, this study connected only the “culturally-attuned motivational and reinforcement SMS messages” feature of SMASH with the value of “being seen as a person rather than a patient” [58,61] (Figure 2). This feature delivers motivational and reinforcement messages tailored to the patient’s values, beliefs, and short- or long-term life goals [62]. This is arguably an important yet challenging objective for value sensitive design. The shift from hospital- to home-based care could be accompanied by a change in perspective about how individuals are treated. Novel eHealth design approaches could take into consideration recent studies that explored ways to identify, elicit, and communicate about the values of individuals with multiple chronic conditions [82-85].

### Applications and Challenges of Value Sensitive eHealth Design for Self-management

The potential connections described in the previous sections represent only a first step toward a value sensitive approach to the design of eHealth for CVD self-management support. Operationalizing value sensitive design will certainly require more than making one-to-one connections between features and values, mainly because self-management is a naturalistic, dynamic, and complex decision-making process [4,86]. Self-management entails distinct and often conflicting goals [86] (eg, health goals vs personal life goals [87,88]), intricate interactions between different actors (eg, patients, families, caregivers [88,89]), and many influencing factors (eg, skill, motivation, confidence [86]). eHealth must aim to facilitate self-management processes, whether it is by delivering only key information, allowing care customization, or addressing person-specific barriers [88].

Moreover, studies involving patients with multiple chronic conditions have also shown the challenges in the identification and conceptualization of their values [90,91]. For example, a study has shown that values can be explicitly or implicitly stated by patients, be also in conflict in with each other, and extend across several conceptual domains [91]. Therefore, value sensitive design is in itself a complex approach and cannot be expected to account for all the challenges ascribed to eHealth self-management solutions. However, its importance lies in the premise that it aims to maximize the patients’ motivation to engage in their own care. Some of its methodological challenges are worth discussing: first, the required methods for the elicitation and translation of values to eHealth design; second, the strategies to simultaneously personalize eHealth to both self-management needs and patient values; third, the underlying research and development approaches through which the aforementioned challenges can be tackled.

### Elicitation and Translation of Values to Design as a Collaborative Task

The elicitation and translation of values to eHealth design is a task that demands the involvement of multiple stakeholders, including health care providers, patients, and their families [82,83]. The findings of this study represent only hypothetical connections that must be validated in consideration of the key elements of a patient’s work system (ie, the persons, tasks, tools, and surrounding contexts) [89]. For example, studies involving informal (family) caregivers report the feelings of stress and anxiety caused by a patient’s discharge from a hospital [92]. Both patients and caregivers alike expressed the need for more involvement of health care providers in this follow-up process [92]. Although this study identified features that connect with similar values such as “having reliable information and advice,” it is unclear if the conceptualization accurately expresses the interests and needs of informal caregivers. It is necessary to validate all observed connections with the actors that become implicit participants by eHealth design (eg, expert-based support features imply the involvement of clinicians and nurses). At early stages of eHealth development, human-centered [93] or holistic approaches to eHealth [18] could be instrumental for the elicitation and translation of patient values (ie, a consideration of perspectives from diverse stakeholders and scientific disciplines).

### Personalizing eHealth Design to Self-management Needs and Patient Values

The 98 connections suggest different ways in which eHealth design could be personalized to keep patients motivated and engaged in self-management. However, in naturalistic settings, it is necessary to consider many more influencing factors before settling for a personalization strategy. For example, older adult patients, a majority in chronically ill populations, often experience cognitive decline [94], have to deal with comorbidities [95], and might require training in the use of technologies [10]. For these patients, traditional educational strategies tend to be ineffective [94] while high levels of comorbidity decrease their self-efficacy. This study suggests design choices such as providing feedback during self-management performance or those argued before as capable to support sense-making. In short, it could be hypothesized that older adult patients who highly value “feeling confident” will benefit more from features sensitized to such value. This requirement also makes apparent that overarching remote system personalization features are vital for proper and on-the-go personalization to individual cases (eg, as done by the SMART PSMS [43,44] or the SUPPORT-HF intervention [48,49]).

### Research and Development Approaches to Aid Value Sensitive Design

To ensure its successful operationalization, value sensitive design must be integrated with both existing and novel approaches of eHealth research and development. On the one hand, value sensitive design aims to sensitize researchers and developers to value-centered work, from theory to practice and vice versa [96]. On the other hand, what is also needed are underlying approaches that guide the actual design processes of value sensitive technologies. In eHealth, user- or human-centered frameworks stand out as widely accepted practices for development [93]. However, the practical challenges and pitfalls of these approaches are seldomly reported in published literature [97]. Challenges can come in formative, design, and evaluation stages or as recurrent processes [97]. On top of that, to validate value sensitive eHealth, it will be necessary to test the differences in actual effectiveness trials. Methodologies such as the Multiphase Optimization Strategy (MOST) could be most suitable [98]. MOST’s fundamental idea is that interventions should be optimized to meet specific criteria before conducting a large-scale randomized control trial [98]. Given the motivational aim of value sensitive design, eHealth technologies could be optimized based on multiple criteria of self-management engagement or its health-related outcomes.

### Future Work

Future studies in the area of value sensitive eHealth design should seek to explore and confirm the connections made by this study. Primarily, studies could pursue further validation of the value conceptualizations in CVD populations. If validated, future studies could then seek the integration of other values identified in similar populations (eg, other chronic conditions such as diabetes or chronic obstructive pulmonary disease). Similarly, future studies could revise or expand the categorization of eHealth design features proposed by this study (ie, according to what they aim to do or how they try to do it) [23]. Certainly, design work is and should always be context-specific, and so the operationalization of design features even for similar objectives might never be exactly the same. However, by refining value conceptualizations and by clustering specific design features within identifiable categories, new hypotheses and guidelines could be tested in order to advance value sensitive design across different eHealth applications and contexts.

### Strengths and Limitations

The hypothetical connections identified by this study can be debated from multiple perspectives. For instance, there is a number of caveats that concern the clarity and reliability of the inferred connections. To recall, the connections are the result of combining a content analysis performed by the authors of this study with the input received from researchers and designers of 6 of the 10 reviewed technologies. On the one hand, the deductive coding of the content analysis shows that all 3 raters agreed most of the time (65.2%, see Table 2). Additionally, one-third of the time, 2 of 3 raters agreed (27.1%), and for 7.7% of the total pairings, there was no agreement at all. On the other hand, the KALPHA coefficient for all data was low (0.4536; 95% CI 0.4087-0.4978). However, it must be considered that KALPHA is a strict coefficient that accounts for the expected disagreement and not only the observed disagreement [25,31]. Therefore, the measure punishes when agreements were not achieved by the challenging, interpretative task of linking design features—described as best as possible with the available information—and a set of values, which are, by definition, subjective. Despite this, the hypothetical connections brought forward by the study must also be valued in light of the aims of the study, namely that it was not the objective to immediately agree on a characterization of values and their potential contributions. In fact, the reliability and lack of agreement were deemed relatively negligible given that the next objective of the project is to validate the presumed connections with individuals in the target group. Thus, the most obvious limitation that the study confronts is that all inferences are still hypothetical and expert-based. In other words, the connections between design features and values must continue to be tested, refined, and generalized.

### Conclusions

This study identified 98 connections between design features of existing eHealth technologies and a set of empirically validated values of individuals living with a CVD. Although existing eHealth technologies were already found to have design features that could align well with patient values, some values were not frequently addressed. These results shed light on the importance of value sensitive design for future eHealth technologies. By and large, what this study adds are explicit and specific design hypotheses for future study that still require validation but, nevertheless, promise to advance the uptake and effectiveness of eHealth self-management support for individuals with a CVD.

## Appendix

Multimedia Appendix 1 eHealth technologies and design features included in the content analysis.

Multimedia Appendix 2 Self-assessment form.

## Abbreviations

CeHRes: Center for eHealth Research
CVD: cardiovascular diseases
KALPHA: Krippendorf alpha
MOST: Multiphase Optimization Strategy
