# Supplementary material for: Toward the Value Sensitive Design of eHealth Technologies to Support Self-management of Cardiovascular Diseases: Content Analysis
Source: JMIR Cardio. 2021 Dec 1;5(2):e31985. doi: 10.2196/31985 (PMC8686487; doi:10.2196/31985)
Supplement: Multimedia Appendix 1 [file cardio_v5i2e31985_app1.docx]

# Multimedia Appendix 1

## eHealth technologies and design features included in the content analysis

|  | eHealth technologies | Description | Sources |
| --- | --- | --- | --- |
| 1 | Engage | Engage is a mobile application, a product intended for continuous use over 30 days to stimulate self-care engagement, behavior, and knowledge of patients with heart failure. Engage was designed as a manual data entry tool. In its latest iterations, Engage was designed to support three core activities: LOG, HINT, and GOAL. | Srinivas et al. 2017 |
| 2 | HeartMapp | HeartMapp is a mobile application for engaging patients in self-management, adherence to medication, diet, and physical activity in patients with HF. HeartMapp is proposed to serve as a personal health buddy that includes six main features: 1) assessment, 2) exercises, 3) vital signs, 4) chronic heart failure educational information (HF info), 5) statistics (stats), and 6) medication list and reminders. | Di Sano et al. 2015; Athilingam et al. 2016; Athilingam et al. 2018 |
| 3 | Home and Online Management and Evaluation of Blood Pressure (HOME BP) | HOME BP is a web-based digital intervention, which facilitates home monitoring of BP, medication changes (if patients' BP remains above target) and lifestyle changes (salt restriction, diet, physical activity, and weight-loss and alcohol reduction). It comprises patient-only components but also health care provider support. | Band et al. 2016; Band et al. 2017; Bradbury et al. 2017; Bradbury et al. 2018; Morton et al. 2018 |
| 4 | MedFit | MedFit is a mobile app designed to allow people with CVD to participate in an exercise-based rehabilitation program remotely. The app works in conjunction with a Fitbit Charge HR device and objectively measures PA and heart rate. The app comprises three central sections: exercise, progress, and my healthy lifestyle. | Kuklyte et al. 2017; Prabhu et al. 2017; Duff et al. 2018 |
| 5 | Baek et al.’s Mock-Up | A study aimed to develop a mock-up of a customized mobile health service optimized for patients with CVD through user research and usability testing. A mobile phone app was designed using a mock-up tool. | Baek et al. 2018 |
| 6 | MyHeart: Heart Failure Management | MyHeart aims to decrease the heart failure population’s mortality and morbidity. The system comprises a weight scale and a blood pressure cuff. Besides, bed garments and wearable garments are used to monitor electrocardiogram and respiration during exercise and rest. The user platform groups all sensors, monitoring devices and a personal digital assistant (PDA) which receives data from the monitoring devices and sensors, processes it, and encourages patients in their daily health care. Users are prompted to follow a daily routine divided into morning, exercise and before sleeping contexts. | Villalba et al. 2007, 2008, and 2009 |

|  | Intervention | Description | Sources |
| --- | --- | --- | --- |
| 7 | PATHway (Physical Activity Towards Health) | PATHway aims to empower patients who have CVD to self-manage their CVD risk factors during post hospital-based cardiac rehabilitation (CR). The target behavior of PATHway is to increase levels of physical activity. PATHway uses an internet-enabled and sensor-based home exercise platform as the core component of a personalized, comprehensive lifestyle intervention program. | Walsh et al. 2018; Triantafyllidis et al. 2018 |
| 8 | SMART Personalized Self-Management System (PSMS) | SMART PSMS is a multi-modular system that aims to facilitate support for those suffering from long-term clinical conditions, specifically Chronic Pain (CP), Congestive Heart Failure (HF) and Stroke. The SMART PSMS is a complex intervention, as defined by the Medical Research Council. Together, the components of the system encourage goal setting, enable self-monitoring of symptoms and behavior, encourage the ongoing review of progress towards goals and provide access to quality-assured information about the condition. **Note:** *The features of both the HF and Stroke modules are presented for coding. The interventions uses different devices according to the target group.* | Burns et al. 2010; Mawson et al. 2014; Bartlett et al. 2014; Parker et al. 2014a; Parker et al. 2014b; Mawson et al. 2016 |
| 9 | Smartphone Medication Adherence Stops Hypertension (SMASH) | SMASH aims to improve medication adherence and minimizing clinical inertia in chronic illness. The SMASH system consists of a cellular connected electronic medication tray, that provides reminder signals and smartphone messaging, reminding patients to take their BP medications using a Bluetooth-accessible BP monitor. | McGillicuddy et al. 2012; Sieverdes et al. 2013; McGillicuddy et al. 2013a; McGillicuddy et al. 2013b; Davidson et al. 2015; Chandler et al. 2019 |
| 10 | SUPPORT HF (Seamless User-centered Proactive Provision Of Risk-stratiﬁed Treatment for Heart Failure) | SUPPORT-HF is a home-based system for health monitoring and self-management support in patients with heart failure. The system consists of a mobile application installed on a touch screen Android-based tablet computer connected to a blood pressure monitor and a set of weighing scales using Bluetooth wireless communication. The application includes features that allow participants to review their personal readings via a graphical display, access educational materials and communicate with the study team by pressing a ‘contact me’ button. | Triantafyllidis et al. 2015a and 2015b; Rahimi et al. 2015; Chantler et al. 2016 |

|  | Design feature | Textual description extracted from the sources | Sources |
| --- | --- | --- | --- |
|  | **Engage** | | |
| 1 | Log | This feature facilitates logging information on symptom data (e.g. weight) and self-care behavior (e.g. taking medication). | Srinivas et al. 2017 |
| 2 | Hint / Facts | This feature facilitates reading of practical hints related to heart failure and performing heart failure self-care. The hint function was meant to address self-care knowledge deficits, particularly by suggesting practical ways to relate and apply knowledge. | Srinivas et al. 2017 |
| 3 | Goal | This feature facilitates action-planning daily behaviors based on longer-term goals. | Srinivas et al. 2017 |
| 4 | Progress report | Engage replaces complex data displays with a scrollable summary report with hyperlinks. This feature is a single, consolidated report mimicking the standard scrollable, text-heavy, summary report typical of visit or discharge. | Srinivas et al. 2017 |
| 5 | Deck of cards | Engage was designed for short-term use. In alignment with this goal, it uses the metaphor of a ‘deck of cards,’ with daily deals over the course of a month long period, comprised of LOG, HINT, and GOAL cards. The system considers prior behavior (e.g. trends in logged data) or outside intervention (e.g. by a clinician) to influence the cards selected or dealt over time. Given the identified need for customization, Engage is flexible with respect to the content of the cards and to accommodate both a starter set of default cards as well as the opportunity to create custom card decks (e.g., per patient, per diagnosis, per clinic, etc.). A motivational incentive was added in the form of virtual coins that are earned with each card played and could optionally be redeemed for tangible rewards. | Srinivas et al. 2017 |
|  | **HeartMapp** | | |
| 6 | Assessment | The assessment feature allows patients to enter weight and answer HF symptom questions. The built-in algorithm in HeartMapp uses the information entered by patients on weight and HF symptoms to classify the patients. Patients are classiﬁed into a Green Zone, if HF symptoms are reported as stable or no change in weight; Yellow Zone, if HF symptoms are reported as mild and a weight gain of three pounds in one day or 5 pounds in a week; Orange Zone, if symptoms are moderate and a weight gain of more than 5 pounds in a day; and Red Zone, if HF symptoms require immediate attention. | Di Sano et al. 2015; Athilingam et al. 2016; Athilingam et al. 2018 |

|  | Design feature | Textual description extracted from the sources | Sources |
| --- | --- | --- | --- |
| 7 | Exercises | The exercises feature of HeartMapp includes an animated deep breathing exercise and measures physical activity using distance walked. The breathing exercise is designed to teach patients, using biofeedback, to attain six breaths per minute and offers feedback on their performance. Similarly, the walking exercise encourages users to engage in physical activity by walking for 30 minutes, three to four times a week or as tolerated, and monitors distance walked to provide feedback. | Di Sano et al. 2015; Athilingam et al. 2016; Athilingam et al. 2018 |
| 8 | Vital signs | This feature monitors vital signs in real time using a wearable Bluetooth sensor with built-in algorithms that measures the heart rate, heart rate variability (HRV), respiratory rate, posture, and activity on a daily basis. It stores vital signs data on the user’s phone. | Di Sano et al. 2015; Athilingam et al. 2016; Athilingam et al. 2018 |
| 9 | HF info | This feature enables patients to read and listen to HF educational information on daily self-management, medications, diet, and physical activity, managing other chronic diseases or conditions, and managing feelings (emotions) about HF. The audio-enabled HF educational information in HeartMapp is available anytime in a real-world situation with no bulky papers to carry around and thus proposed to improve knowledge on recommended HF therapies. | Di Sano et al. 2015; Athilingam et al. 2016; Athilingam et al. 2018 |
| 10 | Statistics / Stats | This feature is a graphical module that displays trends on the patient's weight, HF symptoms, vital signs, physical activity, and deep breathing exercise performance. | Di Sano et al. 2015; Athilingam et al. 2016; Athilingam et al. 2018 |
| 11 | Medication list and reminders | This feature includes an editable personalized medication list that reminds patients of their medication schedule and demands an entry to indicate medication taken or reason for missing the medication. | Di Sano et al. 2015; Athilingam et al. 2016; Athilingam et al. 2018 |
| 12 | Clinical team module | The team module is accessed by the clinical team through any computer connected to the Internet. A Web interface presents a list of the patients that the caregiver/doctors can see. After selecting the patient, using a drop down menu the interface retrieves the data from the database and draws easy to interpret curves about the variable of interest. | Di Sano et al. 2015 |

|  | Design feature | Textual description extracted from the sources | Sources |
| --- | --- | --- | --- |
|  | **Home and Online Management and Evaluation of Blood Pressure (HOME BP)** | | |
| 13 | Self-monitoring of BP | In the HOME BP website patients can access several sessions. The first two are ‘An introduction to HOME BP’ and ‘Learning to monitor BP at home’. After completing these, patients are advised to take morning BP readings for 7 days each month. Patients are instructed on how to correctly undertake self-monitoring to promote patient self-efﬁcacy. Training on how to use the Omron M3 monitor is provided through HOME BP online using a demonstration video. | Band et al. 2016; Band et al. 2017; Bradbury et al. 2017; Bradbury et al. 2018; Morton et al. 2018 |
| 14 | (Automated) e-mail reminders | Patients are sent email prompts to engage in target behaviors (log on, collect BP monitor, collect prescription, monitor BP, enter BP readings), which are followed up by the HOME BP supporter when the behavior is not enacted within a specified period. | Band et al. 2016; Band et al. 2017; Bradbury et al. 2017; Bradbury et al. 2018; Morton et al. 2018 |
| 15 | Automated feedback on BP and medication titration / changes (via health care provider) | After the seven readings are entered in to HOME BP online, the mean BP is calculated and feedback provided to the patient and the health care practitioners according to a trafﬁc light system. If BP was very high (red) or very low (blue), they are told to contact their GP. If BP was above target (amber), they are told their prescriber will contact them about a medication change. In that way, the readings are used to inform the medication titration procedure. | Band et al. 2016; Band et al. 2017; Bradbury et al. 2017; Bradbury et al. 2018; Morton et al. 2018 |
| 16 | Education about medication titration | Information provided in HOME BP aims to be motivating, providing strong evidence for the benefit of titrating medications, and addressing potential concerns about unwanted side effects. | Band et al. 2016; Band et al. 2017; Bradbury et al. 2017; Bradbury et al. 2018; Morton et al. 2018 |
| 17 | Optional lifestyle changes | Nine weeks after the intervention begins, participants have the option of choosing an online session to support lifestyle change to help control their BP, specifically weight management, salt reduction, healthy diet, and physical activity or alcohol reduction. Participants were alerted by email when this became available, and saw an option to view the healthy lifestyles session each time they logged on to HOME BP. The rationale for providing these options 9 weeks after the intervention begins is to avoid making multiple behavior changes until participants have an opportunity to form habits around the key target behaviors of self-monitoring and medication adherence. | Band et al. 2016; Band et al. 2017; Bradbury et al. 2017; Bradbury et al. 2018; Morton et al. 2018 |

|  | Design feature | Textual description extracted from the sources | Sources |
| --- | --- | --- | --- |
| 18 | Behavioral support (via health care provider) | Optional additional behavioral support for self-monitoring and lifestyle modifications is available to all patients via the practice support providers, who are trained in applying the CARE (Congratulate, Ask, Reassure, Encourage) approach. Additional support is restricted to a maximum of six face-to-face or telephone support sessions. | Band et al. 2016; Band et al. 2017; Bradbury et al. 2017; Bradbury et al. 2018; Morton et al. 2018 |
|  | **MedFit** | | |
| 19 | Exercise | There are three options related to the Exercise section. Exercise Class brings the user to the list of guided exercise classes that are personalized based on the evaluation of the classes performed earlier. The Log Activity section allows the user to manually log any exercise or physical activity that has been done outside of the application (e.g. swimming). The Test Yourself option allows the user to evaluate their progress using internationally accepted standard physical activity health tests. | Kuklyte et al. 2017; Prabhu et al. 2017; Duff et al. 2018 |
| 20 | Progress | The progress section of the app contains user feedback displayed in charts and graphs so that the users can track their progress over time, for example, track step count. | Kuklyte et al. 2017; Prabhu et al. 2017; Duff et al. 2018 |
| 21 | My healthy lifestyle | This section provides tips and recommendations on lifestyle factors such as healthy eating, alcohol consumption, physical activity, stress management, medication adherence, smoking cessation, and sexual functioning. | Kuklyte et al. 2017; Prabhu et al. 2017; Duff et al. 2018 |
| 22 | Social interaction | MedFit seeks to encourage social interaction through a ‘MedFit group’ where performance can be compared. | Kuklyte et al. 2017; Prabhu et al. 2017; Duff et al. 2018 |
| 23 | Notifications | MedFit provides notifications to help initiate and maintain the behavior change. They provide encouragement and support to users to reach their physical activity goal. | Duff et al. 2018 |
| 24 | Contact us | MedFit provides a technical support number and information. | Duff et al. 2018 |
|  | **Baek et al.’s Mock-up** | | |
| 25 | My health | This feature facilitates checking one’s comprehensive health status. | Baek et al. 2018 |
| 26 | Daily mission | This feature facilitates checking one’s daily mission, as prescribed by a physician. | Baek et al. 2018 |
| 27 | Health information | This feature facilitates finding the health information one wants, which can be tailored to one’s disease. | Baek et al. 2018 |
| 28 | Health questionnaire | This feature sounds an alarm for a new questionnaire survey for participants to fill in. | Baek et al. 2018 |
| 29 | Self-management | In this feature, users can input a value (e.g. blood pressure 120/80) into the app, and then view the value trend via a graph. This can be done for values of blood pressure, blood sugar tests, and body weight. | Baek et al. 2018 |

|  | Design feature | Textual description extracted from the sources | Sources |
| --- | --- | --- | --- |
| 30 | Diary | This feature facilitates choosing an appropriate icon based on one’s daily moods and symptoms. | Baek et al. 2018 |
|  | **MyHeart: Heart Failure Management** | | |
| 31 | Morning context | The morning context lasts from 8 am to 12 am. When users are prepared, they take the PDA and press ‘Start morning activities’. The morning routine starts with a questionnaire about the quality of the sleep. Afterwards, the user is reminded to take his medication. Then, the UI assistant lets the user into the measurement of the blood pressure and the weight. Both measurements will be sent automatically to the patient device through Bluetooth. | Villalba et al. 2007, 2008, and 2009 |
| 32 | Post morning context | Following the morning routine, the UI assistant guides the user through the body vital signals on how to use the wearable garments. The garment measures ECG and respiration. The quality of the signal is improved when the user is relaxed. Thus, the UI device advices the user to stay relaxed and it discards the signal when it is not good enough. With it, the morning routine is finished. | Villalba et al. 2007, 2008, and 2009 |
| 33 | Exercise measurement | The user can perform a proposed physical exercise to improve his/her heart and physical condition. The user wears the garment for the duration of the physical activity. Before starting the exercise, the device will assure that the user is in a good condition to proceed with the exercise combining all signals and information from all the inputs (e.g. weight increase and answers to questionnaires). During the exercise, which can never be longer than 6 minutes, the device gives feedback to the user and controls through algorithms the adequacy of the exercise. | Villalba et al. 2007, 2008, and 2009 |
| 34 | Evening context | The last interaction with the system occurs in the evening, before going to sleep. Users will be asked some questions about their general wellbeing and will indicate the UI device that they are going to bed. Then, ‘the bed’ will monitor users during the night. | Villalba et al. 2007, 2008, and 2009 |
|  | **PATHway (Physical Activity Towards Health)** | | |
| 35 | Individually tailored exercise program | Before an individual starts to use PATHway, a consultation on how to use the system is held with the participant, whereby they consider the recommended exercise prescription and agree upon it. PATHway presents patients with various ways of achieving their exercise prescription, including ExerClasses and ExerGames where the patients’ movements are captured by MS Kinect camera, and heart rate is sensed by means of the MS Band 2. | Walsh et al. 2018 |

|  | Design feature | Textual description extracted from the sources | Sources |
| --- | --- | --- | --- |
| 36 | On-screen positive reinforcement | PATHway provides visual cues on exercise accuracy during an ExerClass through different levels of positive reinforcement. At the end of a session, a summary is presented including exercise duration, average HR, and exercise accuracy. | Walsh et al. 2018; Triantafyllidis et al. 2018 |
| 37 | Multiplayer class | PATHway can provide a multiplayer class of up to four people, which allows participants to speak before, during, and after exercise to facilitate social interaction. | Walsh et al. 2018 |
| 38 | Calendar for events/exercise | An online calendar allows patients to create, promote, and respond to local and personal events, and to invite others to join. | Walsh et al. 2018 |
| 39 | Lifestyle assessment questionnaire | As part of its Health behavior change program, PATHway first provides questionnaires to assess whether a lifestyle change should be recommended to the participant. | Walsh et al. 2018 |
| 40 | Good habits visualization | The results of the lifestyle assessment lead to the ‘Good habits visualization’, which is a visual display of all risk factors allocated into categories (well done, room for improvement, make a change) to exhibit what behaviors are deemed priorities from the lifestyle assessment. | Walsh et al. 2018 |
| 41 | Behavioral change assessment | Following the ‘Good habits visualization’, four questions are used by the system to assess a participants’ readiness to change a selected behavior. This method is known as the ‘traffic light assessment’. This assessment assigns the participant to a group appropriate to their behavior change stage. | Walsh et al. 2018 |
| 42 | Goal recommendation | Following the behavioral change assessment, a goal is suggested by the PATHway system. For example, if they are deemed as ‘ready for change’ they are shown the text ‘Excellent! You are ready to start-here is an exercise goal just for you’. Alternatively, if deemed ‘ambivalent toward change’ they are prompted through the PATHway flow to engage in decisional balance activities. Finally, if deemed to ‘not be ready for change’ they are directed to engage with the provided educational and support content and consider a change in the future. | Walsh et al. 2018 |
| 43 | Behavior change notifications | To maintain consistent engagement with PATHway even when not actively using the system, tailored behavior change notifications are also delivered to the participant via SMS or email depending on participant preference. These messages are linked to the patient-chosen health behavior goals and are rule-based messages linked to participants’ weekly performance. Targeted lifestyle content is also delivered irrespective of performance. | Walsh et al. 2018 |

|  | Design feature | Textual description extracted from the sources | Sources |
| --- | --- | --- | --- |
| 44 | User dashboard | The wrist-worn HR/physical activity monitor data is synthesized and displayed via the participant’s personalized dashboard. This provides accessible summaries of physical activity data, which can also be accessed by health care professionals. All information pertaining to an individual’s exercise prescription is available on the landing page of the dashboard (e.g., minutes of physical activity achieved so far, how many Exerclasses are left to do). More detailed information on each exercise session can also be viewed (e.g., heart rate ranges during a session and analysis by warm-up, aerobic, and cool down phases). | Walsh et al. 2018 |
| 45 | My healthy lifestyle | MyHealthyLifestyle is a content-based module of PATHway included in the user dashboard. The module gives patients further information on CVD healthy lifestyle behaviors (i.e., physical activity, smoking cessation, alcohol reduction, healthy eating, stress management, and medication adherence). This feature includes basic and advanced content, as well as ‘Ask the expert’ and ‘Peer’ videos. | Walsh et al. 2018 |
| 46 | Technical support | PATHway also provides a helpline number and a support website to address any issues encountered by the user. | Walsh et al. 2018 |
|  | **SMART Personalized Self-Management System (PSMS)** | | |
| 47 | My HomeScreen | The system can be used in the home (via the ‘home hub’, a touch-screen computer interface) and has specific goals and exercises that can be carried out within the home/domestic environment. | Mawson et al. 2014; Bartlett et al. 2014 |
| 48 | Information and advice | The system’s information and advice section contains educational material about the disease and quizzes which aim to test knowledge and provide feedback to the patient, to increase awareness. | Mawson et al. 2014; Bartlett et al. 2014 |
| 49 | My stroke | The system is integrated into the stroke care pathway during the final weeks of community rehabilitation. It allows the customization and personalization of the system for each stroke patient. | Mawson et al. 2014; Mawson et al. 2016 |
| 50 | My goals (Stroke) | The system has a library of exercises linked to ‘‘life goals’’ which users are encouraged to establish in collaboration with a health care professional. | Mawson et al. 2014 |
| 51 | My exercises (Stroke) | The system enables users to make choices about when to exercise and how to exercise. It aims to help individuals relearn motor behavior by encouraging achievement of personal functional goals and repetition of key motor activities within those goals. | Mawson et al. 2014; Mawson et al. 2016 |

|  | Design feature | Textual description extracted from the sources | Sources |
| --- | --- | --- | --- |
| 52 | Today’s exercises (Stroke) | Prior to carrying out any daily exercises, the system asks the user if they are well and about their mood. Depending on the report, an action screen follows to indicate what the user should consider.  If the user indicates that they are unwell, the following screen tells them why they are on this screen and asks them to check for signs and symptoms of serious illness including possibly having another stroke. If they then indicate that they are suffering a sudden onset/exacerbation of one or more of these symptoms, they are encouraged to call an emergency number. If they are unsure and want to check again or have pressed the wrong button by mistake, they can go back to the previous screen. | Mawson et al. 2014; Parker et al. 2014a |
| 53 | Upper-limb rehabilitation (Stroke) | The system monitors and tracks the upper arm rehabilitation movements in real time. It allows motion patterns to be identified, analysed and corrected by both the patient and the therapist. The program is designed to enable recording and playback using an avatar presentation and provides qualitative knowledge of performance describing the characteristics of performance and knowledge of results describing the result of a performance and summary feedback (over a period of time) in chart. | Parker et al. 2014b |
| 54 | Walking re-education and foot placement (Stroke) | The system enables collection of kinematic and usage data. The system provides rewarding feedback as a result of tracked activity. For instance, via motivational feedback on the achievement of walking skill (tracked via ankle and insole sensors). | Mawson et al. 2014; Parker et al. 2014a |
| 55 | My daily plan (HF) | The system allows the input of blood pressure and weight measurements (for HF patients). Patients can also answer symptom questions and produce a daily plan of activities. | Bartlett et al. 2014 |
| 56 | My progress (HF) | The system allows users to get graphical feedback on weight, blood pressure, symptoms and activity levels. | Burns et al. 2010, Bartlett et al. 2014 |
| 57 | Walking intervention (HF) | In the HF module, the system facilitates a walking intervention that aims to increase the patient’s physical fitness. Pacing is taught by the system by providing feedback on activity, and showing users weekly plans, highlighting instances of over activity. | Burns et al. 2010; Bartlett et al. 2014 |
| 58 | My maps and travel | The system allows the users to review a map of the day’s walk using GPS (in the HF module). | Bartlett et al. 2014 |
| 59 | My review (HF) | The system allows users to review their activity. In the case of HF patients, to assess their daily walk exercise as ‘Too Hard’, ‘Just Right’, or ‘Too Easy’. Users can also adjust the next day activity. | Bartlett et al. 2014 |

|  | Design feature | Textual description extracted from the sources | Sources |
| --- | --- | --- | --- |
|  | **Smartphone Medication Adherence Stops Hypertension (SMASH)** | | |
| 60 | Feedback on self-monitoring of BP | Patients are asked to take BP measurements every 3 days in the morning and evening. After a measurement, patients receive immediate smart phone feedback of their average BP after each session and can select charts from the app showing cumulative averages across weeks/months compared to threshold lines for BP control. | McGillicuddy et al. 2012; McGillicuddy et al. 2013a; McGillicuddy et al. 2013b; Sieverdes et al. 2013 |
| 61 | Medication tray reminder signals | Patients are asked to take medications within 90 minutes before or after designated times. In the electronic medication tray, when the medication is to be taken, a blinking light from a specific dose activates. If the pill container is not opened, removed and returned in 30 min, a loud chime activates for 30 min. After that, an automated personalized reminder call goes out (and to a significant other if desired). | McGillicuddy et al. 2012, 2013a, 2013b |
| 62 | Culturally-attuned motivational and reinforcement messages (SMS) | Patients receive mobile phone-delivered personalized motivational and reinforcement messages. These are based upon their medication adherence (MA) levels of the previous day (first month) and then every several days after that, via their preferred mode of delivery (text, email, voice mail). The messages are tailored, based upon one’s values, beliefs and short and long-term life goals. | McGillicuddy et al. 2012; Davidson et al. 2015 |
| 63 | Clinical inertia alarms (to health care provider) | Therapeutic or clinical inertia is the failure to respond in timely manner to clinical data that signal that new or increased treatment is required. In SMASH, health care providers receive weekly or bimonthly summary reports of patients’ medication adherence and at-home blood pressures. Providers are notified immediately if at-home blood pressure exceeds predetermined safety thresholds. Collectively, this information is used to make titration changes for the patients and in some cases to schedule office visits. | McGillicuddy et al. 2012; Chandler et al. 2019 |
|  | **SUPPORT HF (Seamless User-centered Proactive Provision Of Risk-stratiﬁed Treatment for Heart Failure)** | | |
| 64 | Self-monitoring | The mobile system enables patients to complete physiological measurements at home through commercially available sensing devices. Possible self-monitoring features are blood pressure, weight, and pulse oximeter. | Triantafyllidis et al. 2015a, 2015b |
| 65 | My health diary | Participants are asked to complete symptoms diary and physiological measurements on a daily basis if possible, but the recommendations are not prescriptive so that the team can learn from participants’ preferences on timing and frequency of measurements. Symptom diaries constitute clinically validated questionnaire-based instruments in order to assess the patient’s health status. Examples of questionnaires are the New York Heart Association (NYHA), the EuroQol, and the Minnesota Living With Heart Failure (MLWHF) questionnaires. | Triantafyllidis et al. 2015a, 2015b; Rahimi et al. 2015 |

|  | Design feature | Textual description extracted from the sources | Sources |
| --- | --- | --- | --- |
| 66 | My readings | For self-tracking purposes, participants have access to an overview of their personal readings, which are presented via a graphical display. | Triantafyllidis et al. 2015a; Rahimi et al. 2015; Chantler et al. 2016 |
| 67 | How to keep healthy | In this section, participants can access educational materials, video clips, and documents. These include animations illustrating what happens when you have HF and a collection of patients’ stories about their experiences of living with HF (extracted from: Healthtalk.org). | Triantafyllidis et al. 2015a; Chantler et al. 2016 |
| 68 | Contact | Patients can contact the study team (clinicians, administrators, engineers, and a social scientist) by pressing a ‘contact me’ button that triggers an email and text messages to the health professional team. | Triantafyllidis et al. 2015a; Rahimi et al. 2015; Chantler et al. 2016 |
| 69 | Alarms and text-based support (from health care providers) | The team can also send text messages to participants. Text messages are used to comment on health status and in-built alerts are issued. In the back-end web-based application authorized clinicians and nurses can (1) view the patient data graphically, (2) add notes for reporting ﬁndings on both the medical status and usability of the system, (3) send personalized messages to the patients, (4) activate or deactivate features of the self-monitoring component for a speciﬁc patient during system run-time operation, and (5) add the required patient demographic, clinical, or technical information. | Triantafyllidis et al. 2015a; Rahimi et al. 2015; Chantler et al. 2016 |
| 70 | Remote system refinements and features activation | The delivery of reﬁnements to the patient’s tablet computer takes place remotely and unobtrusively through an Internet-linked distribution channel. In addition, when appropriate, functional features can be activated or deactivated by the clinicians according to speciﬁc monitoring requirements. As an example, the functional features associated with a technological device that may not be suitable for a speciﬁc patient after a time period can be deactivated remotely by the clinician during system run-time operation. The users are also notiﬁed of any new functionality coming with service reﬁnements, as well as any possible user interface changes, in order to avoid confusion. | Triantafyllidis et al. 2015a; Rahimi et al. 2015 |
