# Supplementary material for: Toward the Value Sensitive Design of eHealth Technologies to Support Self-management of Cardiovascular Diseases: Content Analysis
Source: JMIR Cardio. 2021 Dec 1;5(2):e31985. doi: 10.2196/31985 (PMC8686487; doi:10.2196/31985)
Supplement: Multimedia Appendix 2 [file cardio_v5i2e31985_app2.docx]

# Multimedia Appendix 2

## Self-assessment form

| **MATCHING PATIENT VALUES WITH EHEALTH FEATURES TO SUPPORT SELF-MANAGEMENT OF CVD** | | | | |
| --- | --- | --- | --- | --- |
| In a previous study, different values of importance for patients living with a cardiovascular disease were identified. A *value* is defined as an ideal or interest that a (future) end user of a technology aspires to or has, in regard to the self-management of their condition.  We would like to know if or how an intervention you are familiar with sought to accomplish or contribute to our list of identified values. This is not a quality or effectiveness assessment of the intervention, but rather an analysis that we believe could inspire the design of future interventions and general investigations into value-based design.  **Instructions:** Consider the questions below and fill in the empty cells. Under ‘Response’ you can type a *YES/Y* or a *NO/N*, according to what best represents your opinion. Under ‘Feature(s)’, you can specify your response if applicable.  **Question 1:** *Do you consider that the* ***________________________*** *intervention accomplishes or contributes to any of the* ***patient*** ***values*** listed in the table below*?*  **Question 2:** *When applicable, can you specify which* ***feature*** ***or part of the intervention*** *you consider seeks to accomplish or contribute to the corresponding patient value?* | | | | |
| **#** | **Patient value** | **Definition** | **Response** | **Feature(s)** |
| 1 | To have confidence in your treatment/therapy or the ability to achieve your goals | *Having confidence in your doctor and the treatment he/she prescribes, or having the feeling that you are capable to follow the treatment plan or the ability to achieve your goals.* |  |  |
| 2 | To be seen as a person rather than a patient | *Not constantly feeling that you are a patient with a disease, also still being able to be a human without your illness.* |  |  |
| 3 | To not feel fear, anxiousness or insecurity about your health | *Not having to worry about your physical condition, being provided coping strategies or information that helps feeling safe or less anxious.* |  |  |
| 4 | To feel a sense of autonomy | *Having a feeling of being in control of your life (e.g. being able to make own decisions).* |  |  |
| 5 | To experience social support | *Feeling heard, supported and understood by the people that surround you (e.g. family and friends) and having the feeling you have somewhere/someone to go to when you need a sympathetic ear (e.g. via a virtual coach or a chat).* |  |  |
| 6 | To become/stay healthy | *Maintaining/changing your lifestyle in such a way that new incidents are prevented and you (re)gain your health.* |  |  |
| 7 | To have an overview of information | *Having a central source where you have insight into your data or condition (e.g. measured values or any insights into physical and mental wellbeing and health).* |  |  |
| 8 | To have a low threshold to access health care | *Being helped or treated quickly and easily, at a health care organization or at home. Being facilitated to manage own disease and take actions.* |  |  |
| 9 | To have ‘a big stick’ to accomplish goals or activities (related to health/lifestyle) | *Being motivated or pushed to do or accomplish things, such as your treatment or activities for a healthy lifestyle (e.g. via social pressure).* |  |  |
| 10 | To have reliable information and advices | *Having understandable, relevant information and advice that is scientifically proven and recommended by physicians (i.e. evidence-based information).* |  |  |
| 11 | To have patient-centered care | *Receiving a personal approach in which your opinion and preferences are taken into account (e.g. personalization or tailoring of treatment choices or features).* |  |  |

If you consider that the intervention sought to accomplish/contribute to **patient** **values** that are not listed in the table above, please describe them in the box below. Also, feel free to add any additional comments or remarks.

|  |
| --- |

Thank you for your contribution to our research!
